# Computational Guide to Optimize Electric Conductance in MoS_2_ Films

**DOI:** 10.1021/acsami.5c05099

**Published:** 2025-06-25

**Authors:** Alireza Ghasemifard, Agnieszka B. Kuc, Thomas Heine

**Affiliations:** † Theoretical Chemistry, 9169 TU Dresden, Bergstraße 66c, 01062 Dresden, Germany; ‡ 28414 Helmholtz-Zentrum Dresden-Rossendorf, HZDR, Bautzner Landstraße 400, 01328 Dresden, Germany; § Center for Advanced Systems Understanding, CASUS, Conrad-Schiedt-Straße 20, 02826 Görlitz, Germany; ∥ IBS-CNM, Yonsei University, Seodaemun-gu, Seoul 120-749, Republic of Korea

**Keywords:** MoS_2_, electric conductance, thin films, DFT, transport calculations, 2D platelets

## Abstract

Molybdenum disulfide (MoS_2_) is a high-potential material for nanoelectronic applications, especially when thinned to a few layers. Liquid-phase exfoliation enables large-scale fabrication of thin films comprising single- and few-layer flakes of MoS_2_ or other transition-metal dichalcogenides (TMDCs), exhibiting variations in the flake size, geometry, edge terminations, and overlapping areas. Electronic conductivity of such films is thus determined by two contributions: the intraflake conductivity, reflecting the value of each single layer, and charge transport across these overlapping flakes. Employing first-principles simulations, we investigate the influence of various edge terminations and the overlap between flakes on the charge transport in MoS_2_ film models. We identify characteristic electronic edge states originating from the edge atoms and their chemical environment, which resemble donor and acceptor states of doped semiconductors. This makes either electrons or holes to majority carriers and enables selective control over the dominant charge carrier type (n-type or p-type). Compared to pristine nanosheets, overlapping flakes exhibit lower overall conductance. In the best-performing hexagonal flakes occurring in Mo-rich environments, the conductance is reduced by 18% compared to the pristine layer, while the drop by 46% and 58% is predicted for truncated triangular and triangular flakes, respectively, in S-rich environments. An overlap of 6.5 nm is sufficient to achieve the highest possible interflake conductance. These findings allow for rational optimization of experimental conditions for the preparation of MoS_2_ and other TMDC semiconducting thin films.

## Graphical abstract

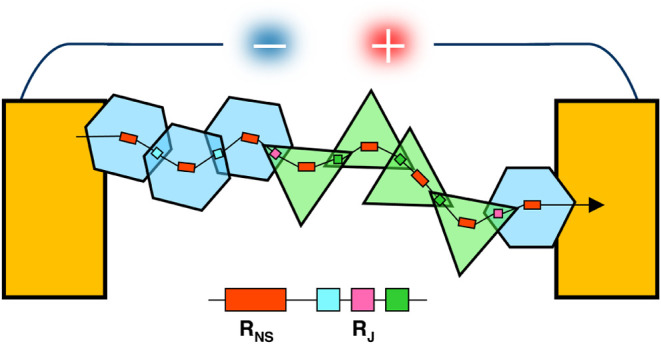

## Introduction

Recent advancements in nanoelectronics have demonstrated the potential of semiconducting nanosheets, produced through liquid-phase exfoliation (LPE), for the fabrication of high-performance printed transistors.^1–5^ In this solvent-based technique, the single- to few-layered nanosheets inevitably exhibit overlap with varying alignments, stacking orders, and diverse edge termination.^6–8^ While LPE is a powerful method to produce nanosheets, the resulting network exhibits a distribution in flake sizes, with mean lengths ranging from a few tens to hundreds of nanometers.^9^ Despite the well-established understanding of the intralayer electronic transport in two-dimensional (2D) semiconducting transition-metal dichalcogenides (TMDCs),^10–14^ a comprehensive investigation of electron transport across flakes at the atomistic scale remains necessary. Recent studies have focused on flake-to-flake transport by examining parameters such as stacking arrangements, dielectric properties, and thickness of graphene and heterostructures of TMDCs.^15–18^ However, to date, there is no detailed atomistic investigation of interflake transport, with edge terminations, flake overlaps, and relative flake orientations. The interlayer transport at the atomic scale provides the missing information needed to bridge the gap to macroscopic models and for the rational design of films with improved application-targeted transport properties.

The bottom-up approach of colloidal chemistry is a powerful method for synthesizing MoS_2_ nanosheets with well-defined sizes.^19–22^ Liquid cascade centrifugation (LCC) effectively separates these nanosheets into distinct size fractions.^23,24^ Nanotomography provides a means to acquire 3D images of nanosheets.^25^ Crucially, when films are formed from liquid-dispersed nanosheets, the flakes deposit with random orientations relative to each other. This inherently leads to overlapping junctions with arbitrary alignments and relative twist angles, in addition to variations in edge termination and overlap area.^3,6^ Understanding the impact of this structural randomness, including the twist between overlapping flakes, is therefore crucial. Through engineering the edge composition of nanosheets, it becomes feasible to obtain MoS_2_ flakes with a precise arrangement of Mo and S atoms.^26–28^ It is possible to preferentially create zigzag (ZZ) edge configurations.^29,29–33^ Importantly, the control over the formation of edges primarily composed of ZZ-Mo or ZZ-S terminations,^26,32,34^ opens up possibilities for tailoring the electronic properties of MoS_2_ flakes.^35–37^ Raju et al.^38^ recently showed that under Mo-rich conditions primarily hexagonal flakes with both ZZ-Mo and ZZ-S edges are formed. Under S-rich conditions, the flake’s morphology changes, gradually decreasing the ZZ-S edges and progressively expanding the ZZ-Mo-S_2_ edges, resulting in a truncated triangular shape. The transition to triangular flakes is driven by the increased stability of ZZ-Mo-S_2_ edges at higher sulfur concentrations, which results in complete suppression of ZZ-S edges. MoS_2_ with ZZ-Mo edges have been successfully synthesized via in situ heating techniques within a transmission electron microscope.^29^ The formation of S–S dimers on Mo edges is promoted by highly sulfiding conditions at elevated temperatures.^39^

In this study, we investigate the influence of edge termination on the conductance of overlapping MoS_2_ flakes, with a focus on how the overlapping region with different lengths, consisting of two stacked MoS_2_ monolayers (MLs) with thermodynamically favorable *H*_h_^h^ and *R*_h_^M^ stackings, influences transport. We focus on ZZ edges, which are the type of edge configurations in exfoliated MoS_2_ flakes. Armchair edges are energetically less favorable and are difficult to obtain through exfoliation. Using the nonequilibrium Green’s function (NEGF) method,^40^ we calculate the electron transmission spectrum and quantum conductance. Our findings show that (i) although overlapping flakes generally have lower conductance than pristine MLs, a greater overlap between layers, specifically a change from ∼1 to 65 Å, increases quantum conductance from 1 to 42% and to 82% (relative to the ML conductance) depending on the edge termination, (ii) an overlap of 6.5 nm allows for maximum conductance between the flakes, (iii) different edge states exhibit preferential charge carriers, favoring either donor- or acceptor-type behavior, leading to n- or p-type semiconductors, and (iv) ZZ-Mo edge (ZZ-S edge) demonstrate constructive (destructive) interference. Our results should assist the rational design of more efficient electronic devices based on printed TMDC flake networks.

## Models

We have investigated overlap MoS_2_ MLs with the thermodynamically dominant *H*_h_^h^ and *R*_h_^M^ stackings (Figure 1a) and different ZZ-edge terminations in individual layers (Figure 1b): pristine ZZ-Mo edge, ZZ-S-Mo (S edge with Mo termination), ZZ-Mo-S_2_ (Mo edge with S_2_ dimer termination), and pristine ZZ-S edge. The *H*_h_^h^ stacking is a 60° twisted variant of the *R*_h_^M^ stacking. Figure 1c illustrates how the morphology of MoS_2_ flakes evolves from hexagonal to triangular shapes with varying chemical species of Mo and S. It also shows the relationship between flake edges and shapes: hexagonal flakes have three ZZ-Mo and ZZ-S edges, truncated triangular flakes have ZZ-S and ZZ-Mo-S_2_ edges, and triangular flakes have only ZZ-Mo-S_2_ edges. We simulated a total of four device models of overlap MoS_2_ MLs with the above-mentioned edge terminations and with varying overlap lengths, *L*_J_ in the case of *H*_h_^h^ stacking (Figure 1d). Devices with mixed-edge terminations of the two layers are also possible, e.g., in *R*_h_^M^ stacking. Furthermore, we investigated two selected twisted bilayer (tBL) MoS_2_ configurations, characterized by twist angles of θ = 27.8 and 38.2°. For structural reasons, twisted flakes have a nonuniform edge termination and overlapping length.

**Figure 1:**
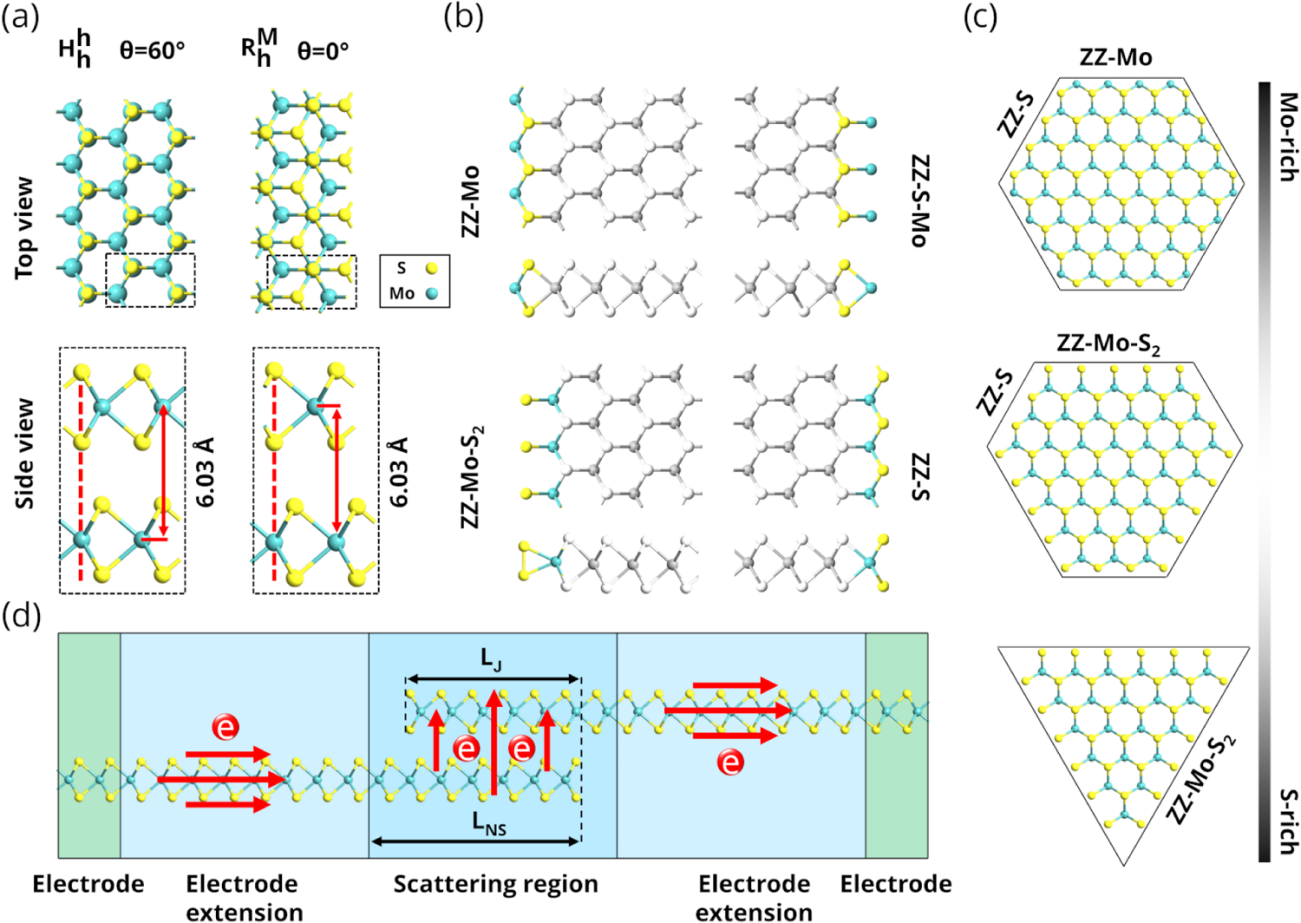
Simulation models: (a) Top and side view of a bilayer (BL) MoS_2_ in the *H*_h_^h^ and *R*_h_^M^ polytypes. Interlayer distance is indicated by the red arrow. Rectangular unit cell, necessary for electron transport simulations, is marked by the dashed lines. (b) Top and side view of possible ZZ-edges: pristine ZZ-Mo-edge, ZZ-S-Mo (S edge with Mo termination), ZZ-Mo-S_2_ (Mo edge with S_2_ dimer termination), and pristine ZZ-S-edge. (c) Schematic illustration of the relationship between flake edge and shape shows hexagonal with ZZ-Mo and ZZ-S edges. Truncated triangular with ZZ-S and ZZ-Mo-S_2_ edges. Triangular with ZZ-Mo-S_2_ edges. (d) Schematic representation of the device configuration. For the exemplary device, ZZ-S edge is used. The device consists of left and right electrodes and electrode extension, which are semi-infinite, and a scattering region. The two lateral and vertical transport paths are denoted by red arrows. *L*_NS_ and *L*_J_ are nanosheet and junction lengths, respectively, and the latter defines the overlap length.

A model device consists of the left and right semi-infinite electrodes, composed of MoS_2_ MLs, and the central transport region, modeled as overlapping MoS_2_ MLs. The transport channel (also called the scattering region) is nonperiodic along the transport direction (from source to drain) and consists of varying *L*_J_, as shown in Figure S1. The in-plane direction normal to the transport is periodic. A vacuum of 20 Å in the out-of-plane direction was used to avoid any interactions caused by the periodic images.

Details of the computational procedures employed, including structural optimization, electronic structure calculations (surface band structure and device density of states (DDOS)), and quantum conductance simulations, are provided in the Methods section. Note that the present approach allows simulations of transport within the coherent limit, neglecting inelastic scattering mechanisms such as electron–phonon interactions.

## Results and Discussion

The simulated electronic properties of ZZ-Mo and ZZ-S shown in Figure 2 (see Figures S2 and S3 for ZZ-S-Mo and ZZ-Mo-S_2_, respectively) are the surface band structure, DDOS, and conductance (*G*) as a function of energy. In addition to the bulk states (dark color), the surface band structure shows the presence of electronic edge states (light green color). A free charge carrier’s response depends on Δ*E* = |*E*_e_ – *E*_VBM,CBM_|, the energy difference between the relevant transport state, i.e., the conduction band minimum (CBM) for electrons or the valence band maximum (VBM) for holes, and the edge state (*E*_e_). Edge states located energetically close to the band edges (within Δ*E* ≈ 4*k*_B_ *T*, where *k*_B_ is the Boltzmann constant and *T* is the temperature) play a dual role. Due to their shallow energy levels, they can be excited and determine the majority carrier type, resembling conventional donor or acceptor dopants. However, due to their spatial localization at the flake boundaries, these same states can also act as scattering centers or temporary traps for mobile charge carriers, thereby reducing charge transmission and restricting overall transport through the material.^41,42^ The 4*k*_B_ *T* energy window is chosen because it represents the accessible states near the band edges relevant for transport at room temperature (*k*_B_ *T* ≈ 25 meV) and corresponds to the energy range typically probed by gate voltage modulation in experimental device characterization.

**Figure 2:**
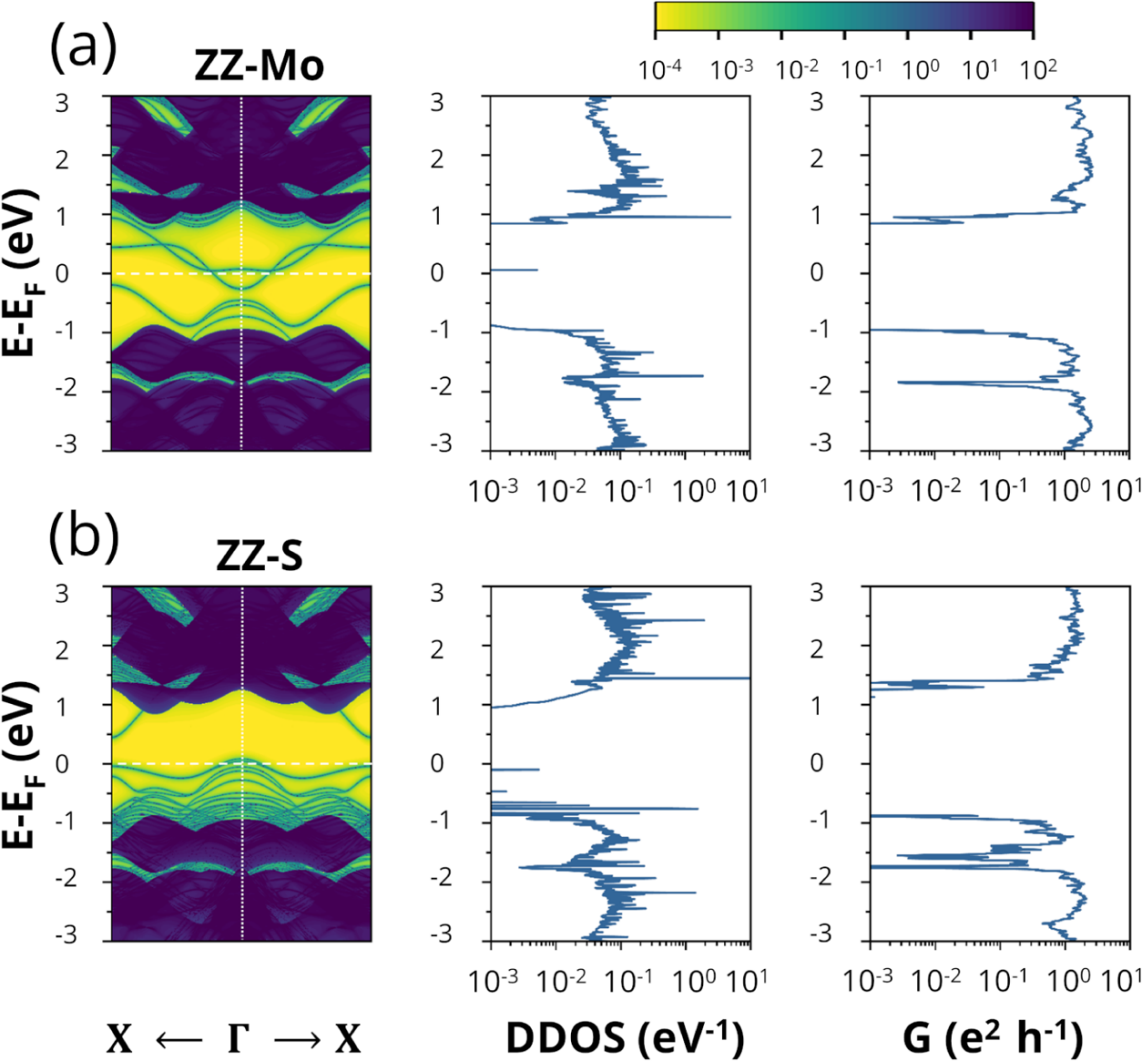
Surface band structure, device density of states (DDOS), and conductance (*G*) as a function of energy for (a) ZZ-Mo and (b) ZZ-S edge termination models. The Fermi level is shifted to zero (white dashed horizontal line). The color bar indicates the weighted DDOS intensity on a logarithmic scale (in units of eV^–1^). Dark blue/purple corresponds to very high DDOS (bulk states), while brighter colors (green/yellow) highlight regions of low DDOS, distinguishing bulk states (darker bands) from prominent edge states within the gap (brighter bands). The spatial localization and dominant orbital character of these edge states are further discussed in the context of Figure 5.

Devices with ZZ-Mo, ZZ-S-Mo, and ZZ-Mo-S_2_ exhibit a high concentration of edge states near the CBM, whereas ZZ-S exhibits them near the VBM. In ZZ-Mo, ZZ-Mo-S_2_, and ZZ-S-Mo, the edge states are likely unoccupied, particularly around the Γ point, and act predominantly as donors, resulting in n-type semiconductors, as evidenced in Figures 2a, S2a, and S3a. In contrast, in ZZ-S, the edge states are more likely occupied and behave as acceptors, leading to a p-type semiconductor, as shown in Figure 2b. A summary of the transport properties of all devices is shown in Figure 3a. Among the investigated devices, the largest conductance (and smallest transport gap) was obtained for ZZ-Mo, while the lowest conductance for ZZ-S and the largest transport gap for ZZ-Mo-S_2_ all with *L*_J_ = 23 Å (cf. Figure 3a).

**Figure 3:**
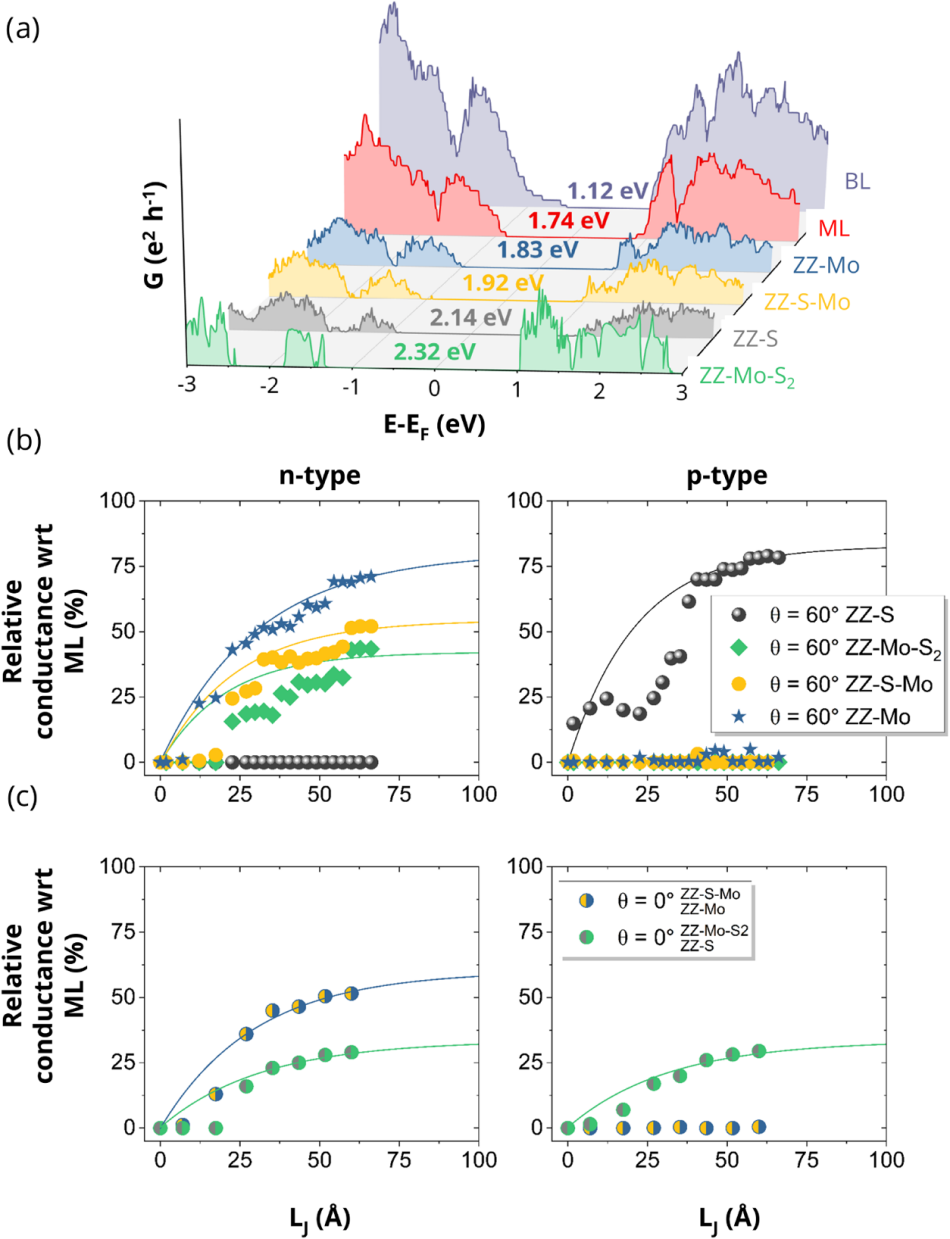
(a) Conductance as a function of energy for all four studied devices, compared to ML and BL. The transport gap of each device is denoted by an arrow and given with color code. The Fermi level is shifted to zero. (b) Relative quantum conductance over Fermi distribution function of *H*_h_^h^ stacking with respect to ML for the states in a ± 4*k*_B_ *T* energy window around VBM and CBM (see Figure S4). Quantum conductance increases as the *L*_J_ increases and reaches saturation at 42, 54, 77, and 82% of the ML value in ZZ-Mo-S_2_, ZZ-S-Mo, ZZ-Mo, and ZZ-S, respectively, with *L*_J_ ≥ 6.5 nm. (c) Quantum conductance of *R*_h_^M^ stacking for mixed-edge types saturates at 58% for edges composed of ZZ-Mo and ZZ-S-Mo and at 31% for ZZ-Mo-S_2_ and ZZ-S. Data points represent calculated values; solid lines are fitted curves to these data, illustrating the trend toward saturation.

Apart from the edge states, a change in the overlap length *L*_J_ of the stacked layers influences the transport. Quantum conductance increases with the systematic increase of *L*_J_ as depicted in Figure 3b,c. *L*_J_ is increased in steps of half the lattice vector (2.75 Å) along the transport direction, see Figure S1. The increase in conductance can be attributed to the increased DOS at the interface, allowing more electrons to be transported. The conductance over a Fermi distribution function in a ±4*k*_B_ *T* energy window around CBM and VBM of ML (see Figure S4) supports the p-type characteristic for ZZ-S and the n-type characteristic for the other edge configurations. Quantum conductance begins to increase for *L*_J_ > 12 Å in n-type devices. As *L*_J_ continues to increase beyond 6.5 nm, the quantum conductance enters the saturation region and remains constant thereafter. Beyond 6.5 nm, the quantum conductance depends on the specific edges with ZZ-S and ZZ-Mo approaching ≈82 and ≈77% conductance of ML, respectively, at large overlaps, as shown in Figure 3b. ZZ-Mo-S_2_ and ZZ-S-Mo approaching 42 and ≈54% conductance of ML at large overlap, respectively.

To investigate the role of stacking and mixed-edge types, we also simulated junctions with *R*_h_^M^ stacking, which represents the other common high-symmetry arrangement. As shown in Figure 3c, by incorporating *R*_h_^M^ stacking with mixed-edge types, we observed similar convergence trends, albeit with different magnitudes, which strongly depend on the constituent edges. Specifically, mixed edges composed of ZZ-Mo and ZZ-S-Mo saturate at 58% of the ML conductance and exhibit only n-type semiconductor behavior, while edges with ZZ-Mo-S_2_ and ZZ-S show both n- and p-type semiconductor characteristics with a significantly lower saturation magnitude of 31%, as depicted in Figure 3c. This result shows that the chemical nature of the edge terminations at the interface is directly related to the saturation conductance. Therefore, while the ML semiconductor provides a reference conductance, the edge configuration and the nanosheet length *L*_NS_ determine the overall transport properties of the stacked structures.

Another factor affecting the flake-to-flake charge transport is the twist angle between the flakes. We considered two distinct regimes based on previous studies:^43,44^ (i) Small twist angles (θ ≤ 13° or θ ≥ 47°), which lead to significant atomic reconstruction forming large domains of stable high-symmetry stackings (predominantly *R*_h_^M^ and *H*_h_^h^). As the characteristic size of these domains typically exceeds the calculated saturation overlap length (6.5 nm), the conductance across such an overlap would be dominated by the local high-symmetry stacking, resembling the untwisted cases already studied. Furthermore, simulating transport across the large supercells required for these small angles combined with sufficient overlap length is computationally prohibitive with DFT-NEGF. (ii) Large twist angles, representing the moiré regime without significant reconstruction. These angles allow us to assess the impact of the twist itself, including minor variations in local stacking and interlayer distance, on the conductance saturation. Therefore, we selected two representative large twist angles, θ = 21.8 and 38.2° (see Figure 4a,b), which fall within this moiré regime and possess reasonably sized unit cells suitable for NEGF calculations. As shown in Figure 4c,d, the calculated interlayer conductance (data points) for these twisted systems changes only slightly compared with the saturation behavior of the untwisted *R*_h_^M^ and *H*_h_^h^ stackings (solid lines), respectively. This indicates that the saturation conductance achievable at larger overlaps (≥6.5 nm) is not significantly affected by these large twist angles compared to the high-symmetry untwisted configurations. Due to the nonuniform overlap resulting from the twist angle in these devices, *L*_J_ is not a single well-defined parameter. Therefore, a maximum value of *L*_J_ within the simulation cell was considered for comparison with the untwisted devices plotted against *L*_J_. The edges in the tBL system are composed of: (i) a mixture of edge types in both layers and (ii) a combination of one edge type in each layer.

**Figure 4:**
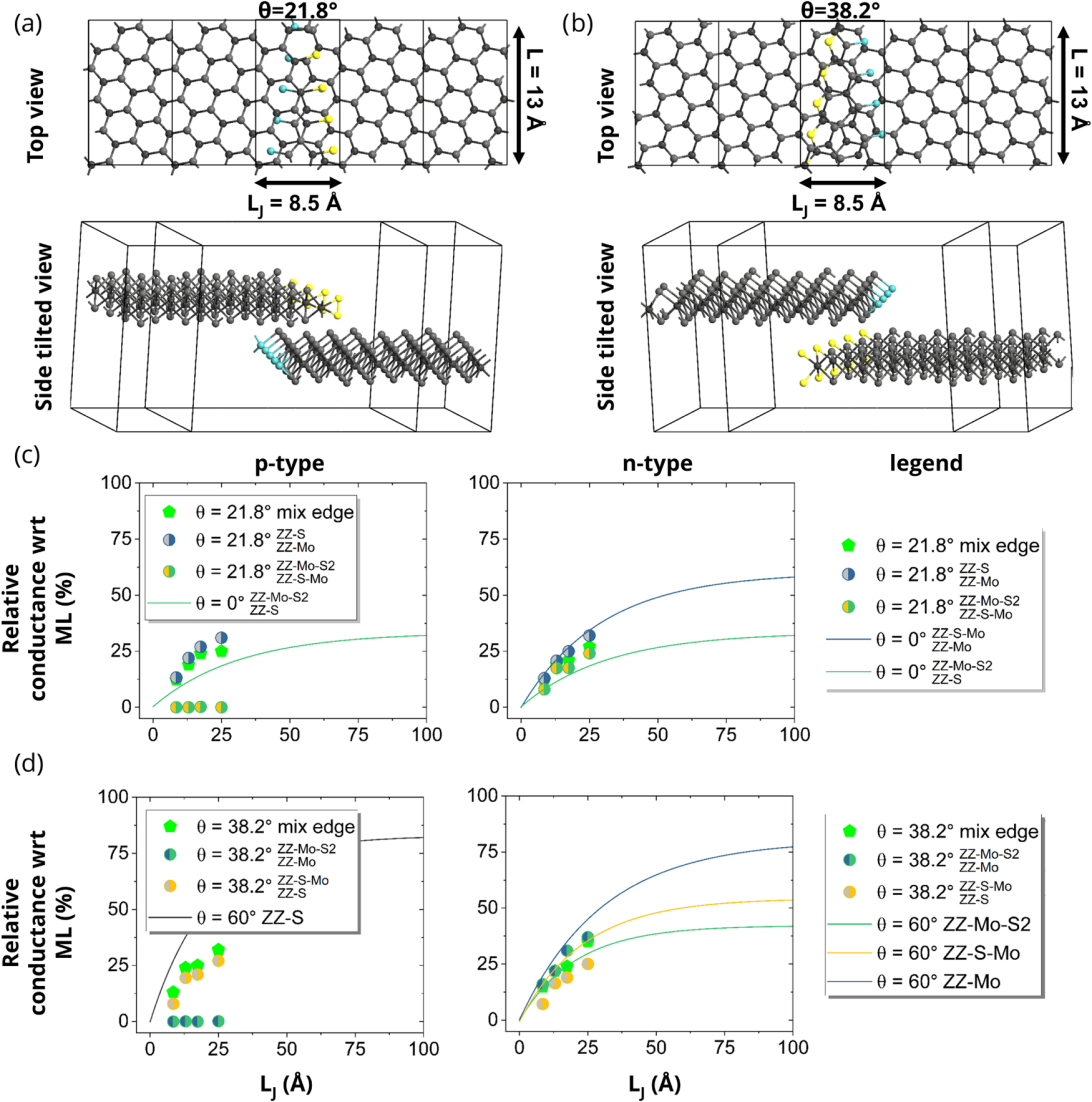
Schematic representation of device configurations with (a) θ = 21.8° and (b) 38.2° with top and side-tilted views. A maximum *L*_J_ value is considered for comparison with those of the untwisted devices. Relative quantum conductance, integrated over the Fermi distribution function within a ±4*k*_B_ *T* energy window around the VBM and CBM, is shown for (c) θ = 21.8° and (d) 38.2°. Data points indicate the relative conductance of a twisted bilayer with a varying *L*_J_, which is compared to the fitted relative conductance of *R*_h_^M^ and *H*_h_^h^ shown by the solid lines in panels (c) and (d), respectively.

Generally, we notice that the size of the transport gap exhibits an inverse dependence on the *L*_J_, gradually decreasing as the overlap length increases (see Figure S5). Also, the interlayer distance (*d*) between overlapping layers influences device performance, with conductance increasing (decreasing) as *d* decreases (increases). A compression of 1 Å from the equilibrium interlayer distance results in a conductance enhancement of 27 and 22% for ZZ-Mo and ZZ-S, respectively. In contrast, expansion by 1 Å leads to a decrease in conductance by 40 and 50% in ZZ-Mo and ZZ-S, respectively (cf. Figure S6).

To investigate the effect of edges and overlap on conductance, we examined the electronic properties in more detail. Figures 5a,b and S7 show the local DOS (LDOS) for each device, with the corresponding wave function in real space, shown in Figure 5c,d. The edge states are dominant inside the junction area in ZZ-Mo, indicating that the ZZ-Mo atoms are primarily localized to the junction and aid in interlayer transport. In contrast, the energy states in ZZ-S are localized at the junction but extend beyond it outside the junction. This is attributed to the localization of S atoms in the electrode extension, which results in the destructive behavior illustrated in Figure 5f. Depending on the type of the edge, different atomic orbitals dominate in the wave function: in ZZ-Mo, states with high Δ*E* are formed from Mo-d_*xy*_ orbitals, while Mo-d_*z*2_ orbitals are responsible for the states with smaller Δ*E*. In ZZ-S, S-p_*z*_ and -s orbitals form states with high and small Δ*E*, respectively. In the cases with mixed Mo-S or S-Mo edges, both S-p_*z*_ and Mo-d_*z*2_ orbitals contribute to the LDOS. Analyzing transmission eigenstates at both the VBM and CBM at the Γ point, we observe constructive transmission states (bright regions) throughout the entire ZZ-Mo device, as shown in Figure 5e. A change from ZZ-Mo to ZZ-Mo-S_2_ under S-rich conditions preserves the constructive interference pattern (see Figure S2b) with a decrease in conductance by 26% with *L*_J_ = 23 Å and 35% with *L*_J_ > 65 Å, indicating that the presence of S_2_ termination in ZZ-Mo-S_2_ has more impact on the overall transport properties in large overlap lengths. In contrast, the right part of the ZZ-S device exhibits destructive transmission states (dark regions) (Figure 5f). The edges are terminated by S atoms, and the corresponding edge states are dominated by S s- and p-orbitals (notably, S p_*z*_ orbitals for interlayer coupling). The inherent nodal structure of p-orbitals (having lobes of the opposite phase) can readily lead to phase cancellations when they couple with orbitals from the opposing flake across the junction. If different electron wave pathways acquire opposing phases upon traversing the interface, they interfere destructively. In ZZ-S-Mo, a different behavior was observed, such as constructive transmission at CBM states and destructive transmission at VBM states, as shown in Figure S3b, which supports the n-type characteristics of ZZ-S-Mo. The transmission eigenstates are not solely dependent on the energy but also on the k-points. While there is a large DOS close to VBM in Figure 5b, this does not necessarily imply bright transmission eigenstates with large amplitudes. Table 1 summarizes the key characteristics and transport properties of various edge types investigated in the present work.

**Figure 5:**
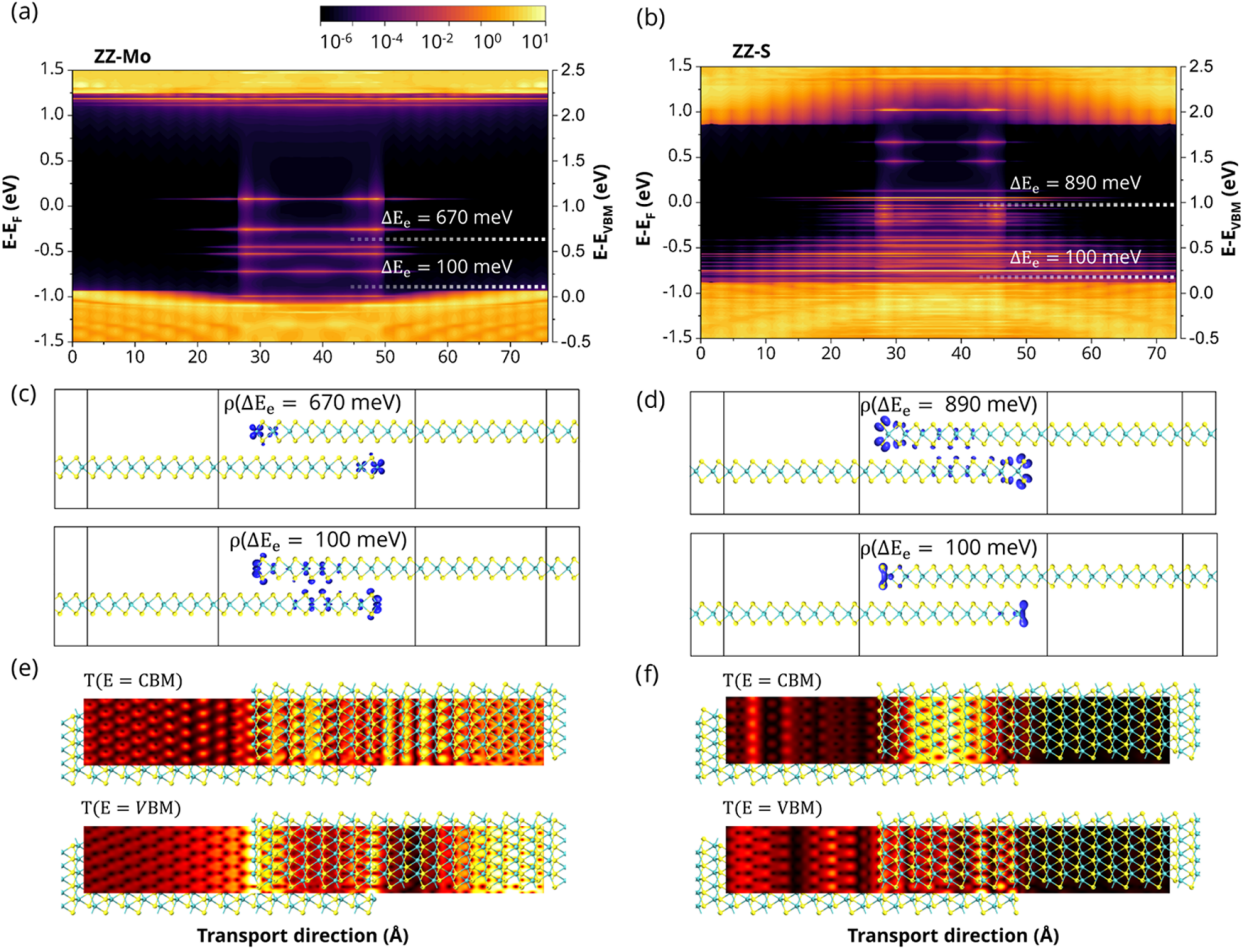
Local density of states as a function of position for (a) ZZ-Mo and (b) ZZ-S, along with the edge states’ wave function spread in real space (c) and (d). The color scale represents the DDOS in units of eV^–1^. The Fermi level is shifted to zero and is shown on the left axis. Transmission eigenstates of (e) ZZ-Mo with constructive interference and (f) ZZ-S with destructive interference both at CBM and VBM energies, and *k*_*a*_ = Γ. The calculated transmission eigenstates have the highest eigenvalue. The amplitude of the transmission eigenstates is on the order of eV^–1/2^ Å^–3/2^. The bright area has the maximum, and the dark area has the minimum amplitude. Charge carriers originate from the lower left lead, travel to the upper right lead in the scattering region, and then return to the reservoir.

**Table 1:** Summary of Edge Type, Device Characteristics, and Transport Properties

edge type	edge states	device type	conductance saturation (%)	transport gap (eV)
ZZ-Mo	donors	n-type	∼77	1.83
ZZ-S	acceptors	p-type	∼82	2.14
ZZ-S-Mo	donors	n-type	∼54	1.92
ZZ-Mo-S_2_	donors	n-type	∼42	2.32

In a 3D network of 2D nanosheets composed of many well-defined, overlapping flakes, the total resistance can be expressed as *R*_Net_ = *R*_NS_ + *R*_J_, where *R*_NS_ ∝ *L*_NS_ represents the resistance of an individual nanosheet (without overlap) and *R*_J_ ∝ *L*_J_ is the resistance of the junction formed by the overlap. As charge carriers traverse the network, they inevitably cross these overlapping junctions, encountering resistance that varies, depending on the edge types of the constituent nanosheets. The overall trend shows that hexagonal flakes are the most interesting models for efficient interlayer transport. Therefore, in order to have maximum efficiency of a device, ZZ edges with either Mo or S are preferred.

## Conclusions

This study provides a computational guide to optimize electrical conductance in MoS_2_ films made of overlapping flakes of commonly observed flake shapes and edge terminations. We show that besides the flake size, the degree of overlap between flakes, the edge termination, and the interlayer spacing are important factors for correctly assessing the transport properties. Our first-principles calculations demonstrate that hexagonal flakes occurring in Mo-rich environments with overlapping ZZ-Mo or ZZ-S edges can achieve the best transport performance. Truncated triangular and triangular flakes with overlapping ZZ-Mo-S_2_ edges occurring in S-rich environments show a poor transport performance. Overlapping flakes decrease overall conductance compared to pristine nanosheets, with conductance drops ranging from 18% in the best-performing system to 58% in the poor-performing system. Increasing the flake concentration to achieve an overlap of more than 6.5 nm does not improve conductance. ZZ-Mo, ZZ-Mo-S_2_, and ZZ-S-Mo edges exhibit constructive interference at the donor states, favoring n-type semiconductors. Conversely, the ZZ-S edge displays destructive interference at acceptor states with p-type semiconductors. Compressing the interlayer distance at the flake overlap interfaces by 1 Å from the equilibrium distance results in a conductance enhancement of 27% for ZZ-Mo and 22% for ZZ-S, while a similar expansion decreases conductance by 40 and 50% for ZZ-Mo and ZZ-S, respectively. The twist angle between the flakes only has a minor impact on the saturation conductance. Thus, a controlled process is needed to construct optimized MoS_2_ films with controlled flake geometry and overlap for achieving the best electrical properties. It is important to note that the focus of this study was on pristine and specific nonoxidized edge terminations characteristic of MoS_2_ under controlled synthesis or exfoliation conditions. In many practical applications, exposure to ambient air can lead to edge oxidation, which is known to significantly alter the electronic properties of MoS_2_.^45–47^ A detailed investigation of the impact of various oxygen-terminated edges is a subject for future studies.

## Methods

### Details of Computational Methods

All systems were fully relaxed using LAMMPS code^48^ with Reax^49–52^ force field (ReaxFF) with a maximum force component of 0.1 eV/Å and pressure of 10 kbar, as obtained from our previous work on BL MoS_2_.^43,44^ The atomic positions were subsequently reoptimized with ReaxFF after creating different types of edges to allow for edge reconstruction. To accurately model the formation of S dimers at the edge of ZZ-Mo-S_2_, a process not inherently captured by the ReaxFF parametrization (see Figure S8), further optimization was carried out using density functional theory (DFT). The twisted bilayer MoS_2_ structures with selected twist angles (θ = 21.8 and 38.2°) were generated by relative rotation of the layers and full relaxation using the ReaxFF as detailed in our previous works.^43,44^ These relaxed structures were used for transport calculations. DFT optimization was performed as implemented in QuantumATK S-2022.03 package,^53^ using the Perdew–Burke–Ernzerhof (PBE) functional,^54^ vdW D3 correction,^55^ and double-ζ polarized pseudopotentials.

Electronic structure calculations (device density of states, DDOS, Local Density of states, LDOS, and surface band structure) were performed employing the DFT+NEGF framework. The DFT part used the PBE functional and pseudo-Dojo pseudopotentials.^56^ A dense 11 × 1 × 199 in *a*- (periodic), *b*- (vacuum), and *c*-direction (transport) Monkhorst–Pack *k*-point grid and 45 hartree density mesh cutoff were used. The DDOS *D*(*E*), representing the states in the central scattering region coupled to the electrodes, is calculated from the trace of the partial spectral density matrices *A*_L_(*E*) and *A*_R_(*E*) originating from the left (L) and right (R) electrodes, respectively:

1 $$D\left(E\right)=\mathrm{Tr}\left[A_{L}\left(E\right)+A_{R}\left(E\right)\right]$$

$A_{\alpha }\left(E\right)=G\left(E\right)\Gamma_{\alpha }\left(E\right)G^{†}\left(E\right)$ for α = L, R. Here, $G\left(E\right)={\left[\mathrm{ES}-H-\Sigma_{L}\left(E\right)-\Sigma_{R}\left(E\right)\right]}^{-1}$ is the retarded Green’s function of the device region (*H* and *S* are the Hamiltonian and overlap matrix elements, respectively. Σ_α_ is the self-energy due to coupling with electrode α), and Γ_α_(*E*) = *i*[Σ_α_(*E*) – Σ_α_^†^(*E*)] are the coupling matrices (broadening functions) representing the coupling strength to the electrodes. The local density of states *D*(*E*, **r**) provides the spatial distribution of the total DOS and can be calculated from the Green’s function within an atomic orbital basis {ϕ_*i*_(**r**)}, as

2 D(E,r)=∑ij−1πIm[Gij(E)]ϕi(r)ϕj(r)

The total DDOS *D*(*E*) is the integral of *D*(*E*, **r**) over all space. The surface band structure is obtained by plotting the DDOS *D*(*E*, *k*_a_) as a function of energy *E* and the wave vector *k*_a_ along high-symmetry directions in the 1D Brillouin zone corresponding to the periodic *a*-direction transverse to the transport.^57^ This allows bulk states to be distinguished from edge-localized states. DOS was calculated using the tetrahedron method^58^ in order to capture the fine feature of the electronic edge states. The quantum conductance *G*(*E*) was calculated for each device using the same DFT+NEGF framework via the Landauer–Büttiker (LB) approach:^40,59,60^

3 $$G\left(E\right)=\frac{2e^{2}}{h}\mathrm{Tr}\left[\Gamma_{R}\left(E\right)G\left(E\right)\Gamma_{L}\left(E\right)G^{†}\left(E\right)\right]$$

where *e* and *h* are fundamental constants, and the terms G , Γ_L_, Γ_R_ are the same Green’s function and coupling matrices used for the DDOS calculation.

## Supplementary Material



## Notes

Figure S1: Schematic of device models with varying overlap lengths. Figure S2: Electronic properties (surface band structure, DDOS, conductance) for ZZ-S-Mo edge termination. Figure S3: Electronic properties (surface band structure, DDOS, conductance) for ZZ-Mo-S_2_ edge termination. Figure S4: Fermi distribution function used for integrating conductance. Figure S5: Transport gap as a function of overlap length (*L*_J_). Figure S6: Quantum conductance as a function of interlayer distance (*d*) for ZZ-Mo and ZZ-S edges. Figure S7: Local density of states (LDOS) for ZZ-S-Mo and ZZ-Mo-S_2_ edge terminations. Figure S8: Comparison of S-dimer structure at ZZ-Mo-S_2_ edge optimized with ReaxFF and DFT. (PDF)
