# Supplementary material for: Computational Guide to Optimize Electric Conductance in MoS2 Films
Source: ACS Appl Mater Interfaces. 2025 Jun 25;17(27):39595–604. doi: 10.1021/acsami.5c05099 (PMC12257452; doi:10.1021/acsami.5c05099)
Supplement: Supplementary file 1 [file am5c05099_si_001.pdf]

Computational guide to optimize electric conductance in MoS_2 films

Alireza Ghasemifard,[†,‡,¶] Agnieszka B. Kuc,[*,‡,¶] and Thomas Heine[*,†,‡,¶,§]

†*TU Dresden, Theoretical Chemistry, Bergstr. 66c, 01062 Dresden, Germany*

‡*Helmholtz-Zentrum Dresden-Rossendorf, HZDR, Bautzner Landstr. 400, 01328 Dresden, Germany*

¶*Center for Advanced Systems Understanding, CASUS, Conrad-Schiedt-Straße 20, 02826 Görlitz, Germany*

§*Yonsei University and ibs-cnm, Seodaemun-gu, Seoul 120-749, Republic of Korea*

E-mail: a.kuc@hzdr.de; thomas.heine@tu-dresden.de

SUPPLEMENTARY INFORMATION

Supporting Information includes the following:

- Figure SI1: Schematic of device models with varying overlap lengths.
- Figure SI2: Electronic properties (surface band structure, DDOS, conductance) for ZZ-S-Mo edge termination.
- Figure SI3: Electronic properties (surface band structure, DDOS, conductance) for ZZ-Mo-S_2 edge termination.
- Figure SI4: Fermi distribution function used for integrating conductance.
- Figure SI5: Transport gap as a function of overlap length (L_J)
- Figure SI6: Quantum conductance as a function of interlayer distance (d) for ZZ-Mo and ZZ-S edges.
- Figure SI7: Local density of states (LDOS) for ZZ-S-Mo and ZZ-Mo-S_2 edge terminations.
- Figure SI8: Comparison of S-dimer structure at ZZ-Mo-S_2 edge optimized with ReaxFF and DFT.

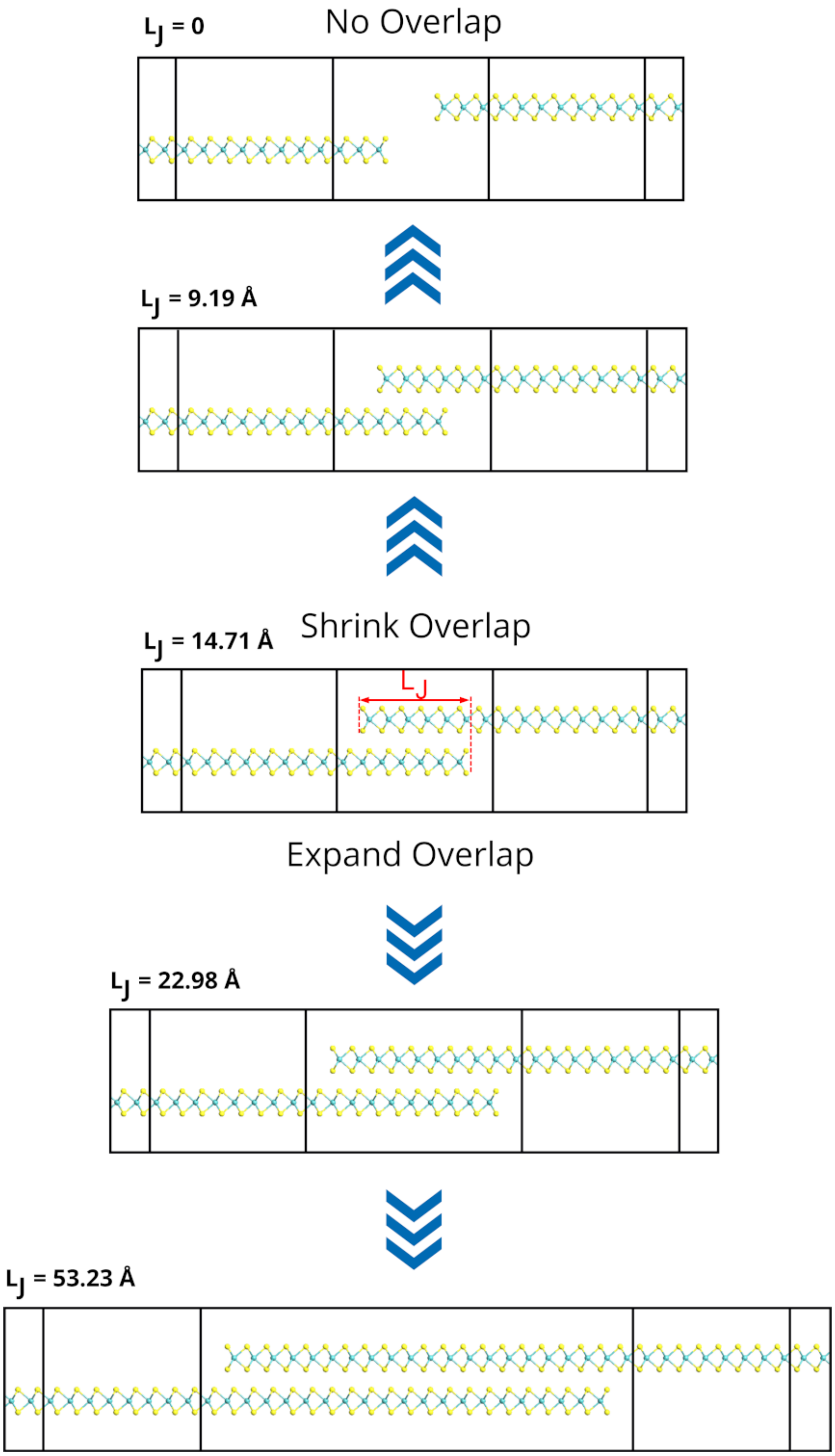

Figure. S1: Schematics of change in overlap length of a device. Starting with the exemplary ZZ-S device with L_J=14.71 Å, it is possible to adjust the overlap length of the scattering region by removing or adding a MoS_2 unit in each layer. This allows contraction or expansion of the scattering region, respectively. The left and right leads, as well as the extension leads, remain unchanged.

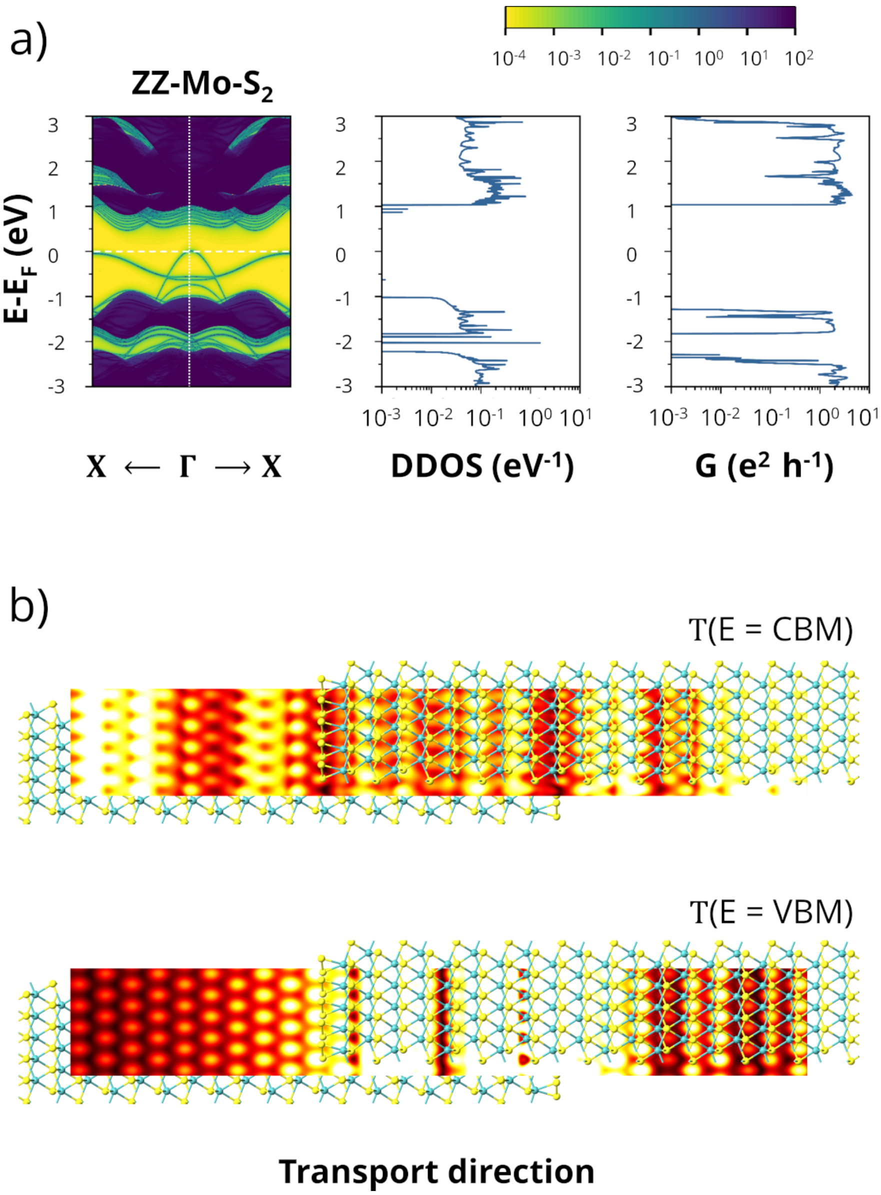

Figure. S2: (a) Surface band structure, device density of states, and conductance as function of energy. (b) Transmission eigenstates at $E = CBM$ and $E = VBM$, with $k_a = \Gamma$ for ZZ-Mo-S_2 device.

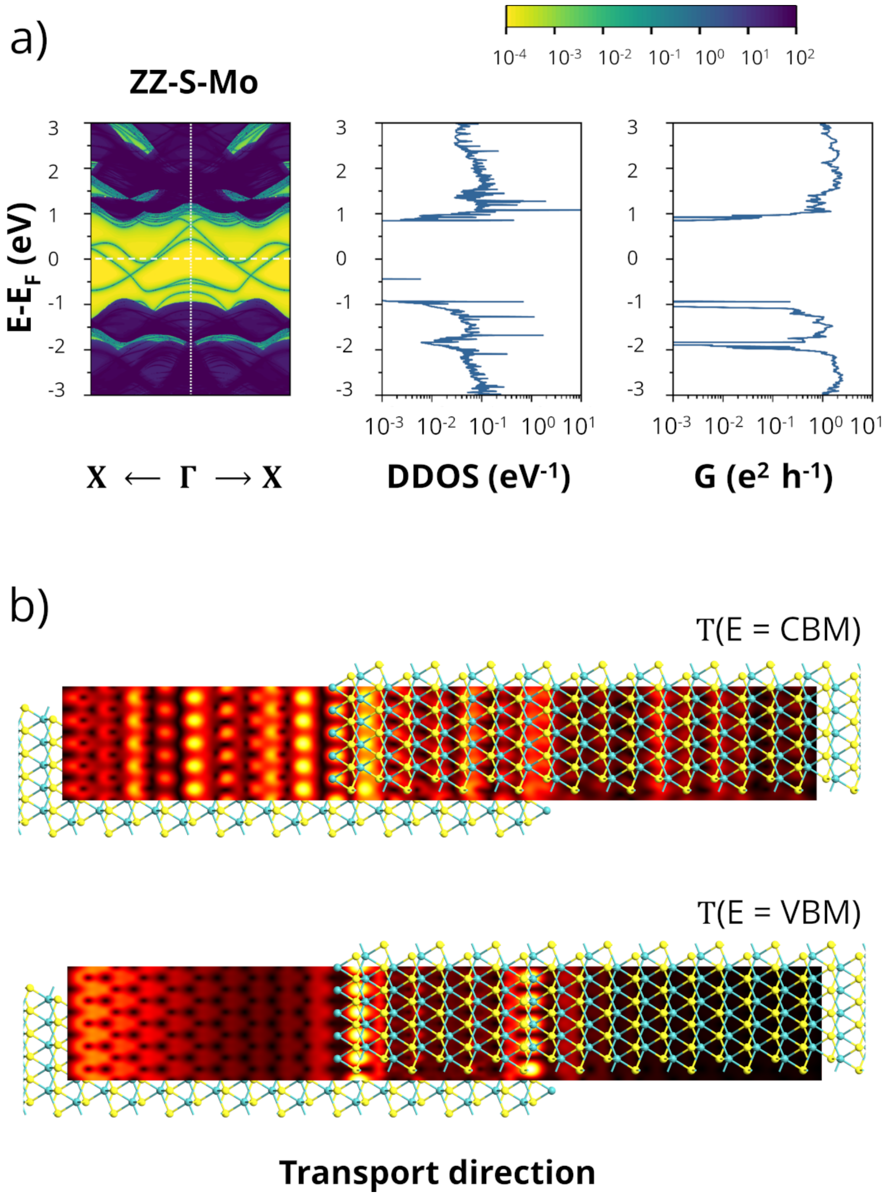

Figure. S3: (a) Surface band structure, device density of states, and conductance as function of energy. (b) Transmission eigenstates at $E = CBM$ and $E = VBM$, with $k_{\rm a} = \Gamma$ for ZZ-S-Mo device.

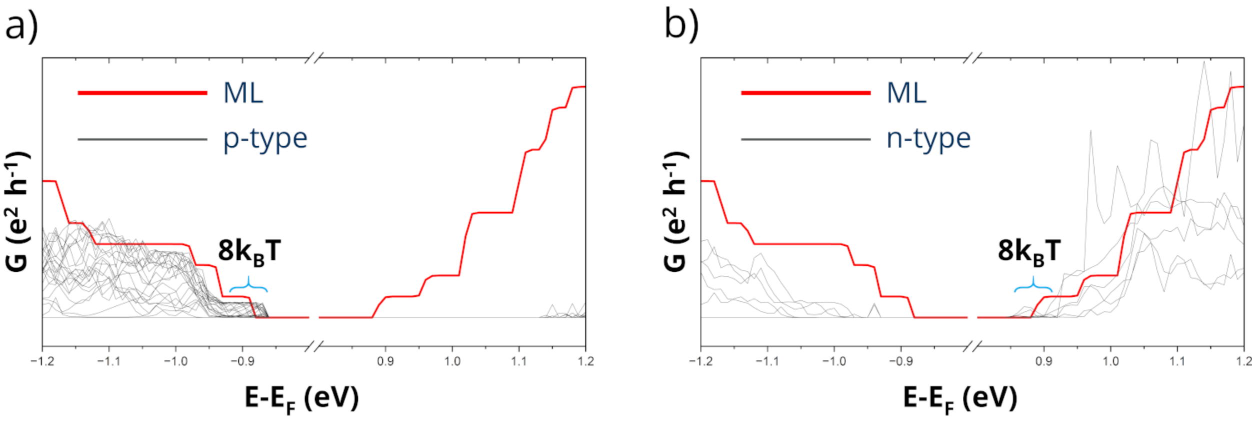

Figure. S4: Conductance as function of energy of ML (red curve) compared to (a) p-type semiconductors (ZZ-S), and (b) n-type semiconductors (ZZ-Mo, ZZ-S-Mo, and ZZ-Mo-S_2). The $\pm 4k_BT$ energy window around CBM and VBM of ML denoted by blue curly bracket.

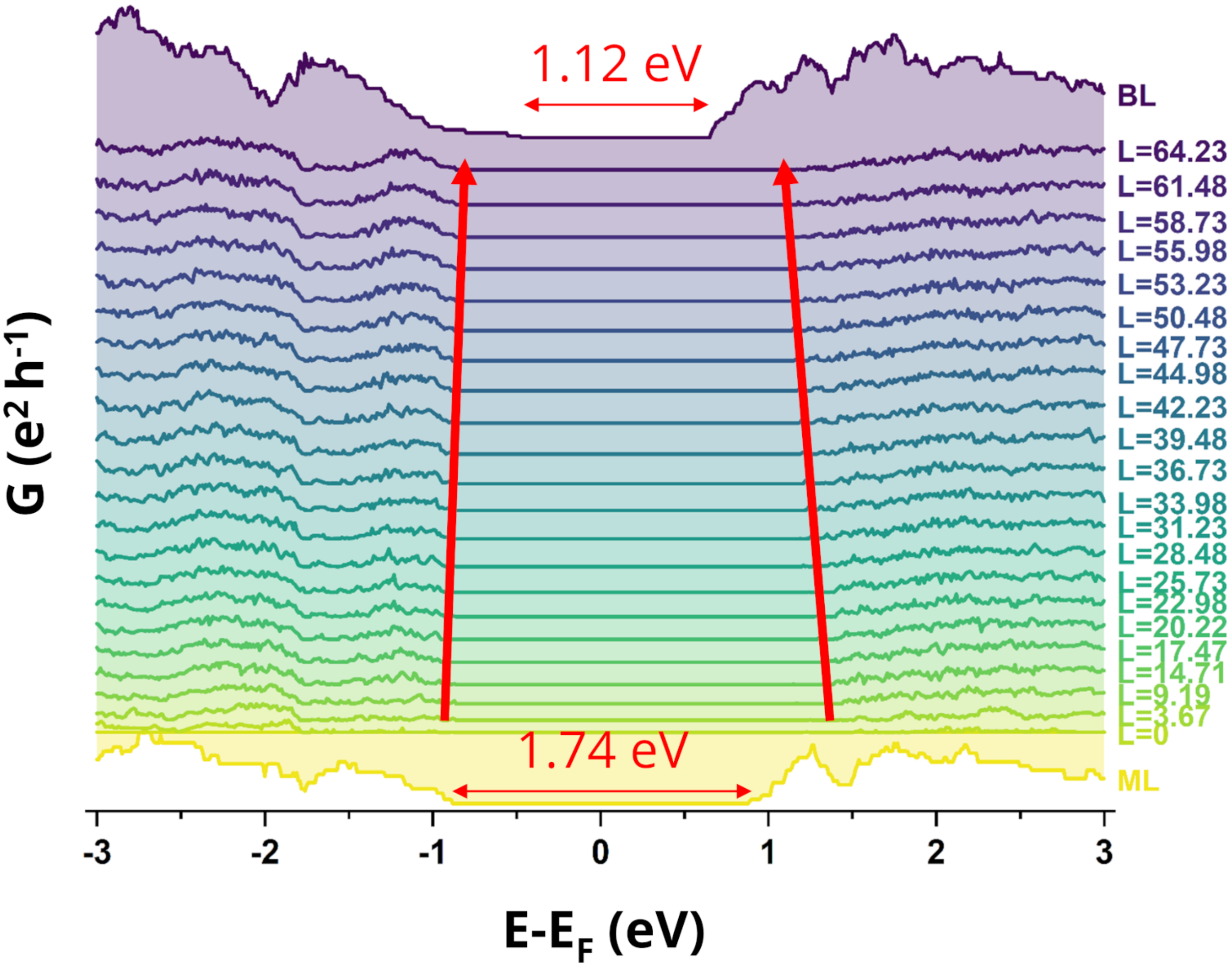

Figure. S5: Conductance as function of energy of ZZ-S device with L_J ranging from 0 to 64.23 Å. The transport gap decreases with increasing L_J.

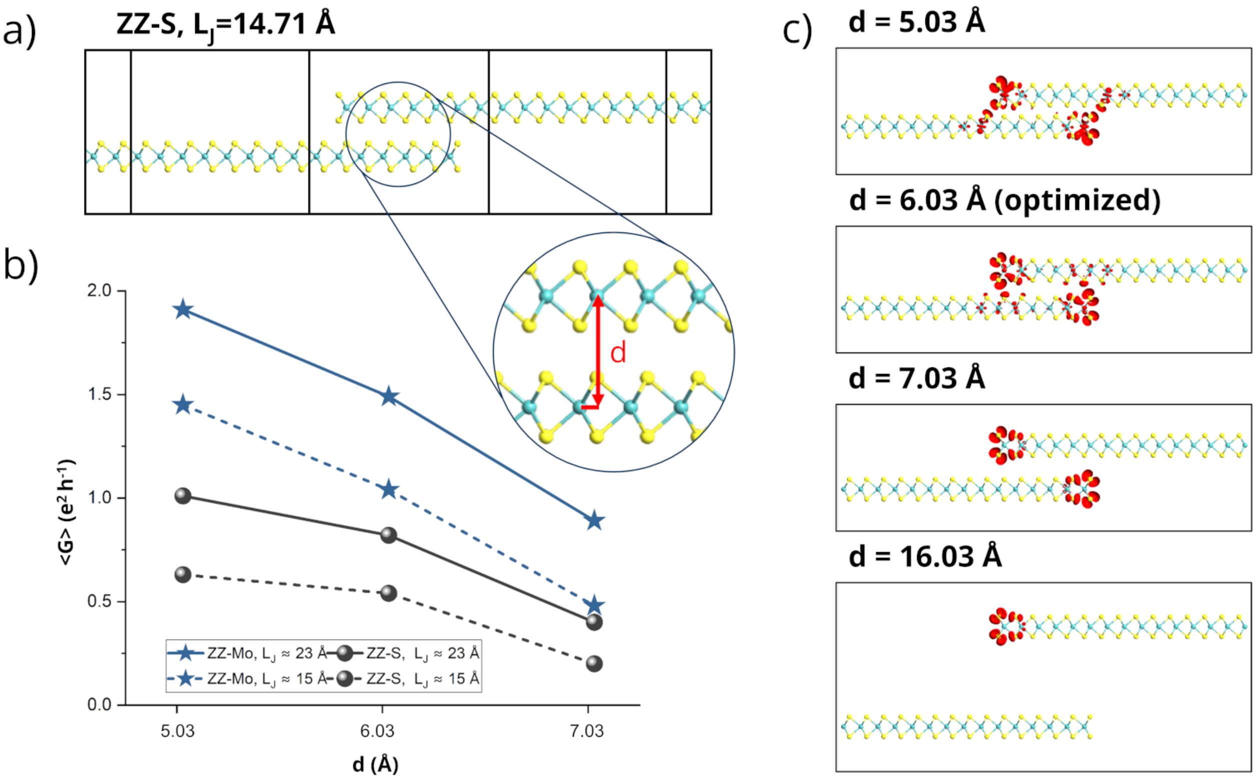

Figure. S6: Analysis of the interlayer distance (d) impact on conductance and Bloch wave functions. (a) Vector representation of d. (b) Average conductance as function of d for ZZ-S and ZZ-Mo. (c) Corresponding wave functions of Blcoh states for specific interlayer distances: $d = 5.03$ Å, 6.03 Å(relaxed case), and 7.03 Å, all with $L_J = 14.71$ Å for ZZ-S at the edges. The Bloch states are selected from edge states near the Fermi energy.

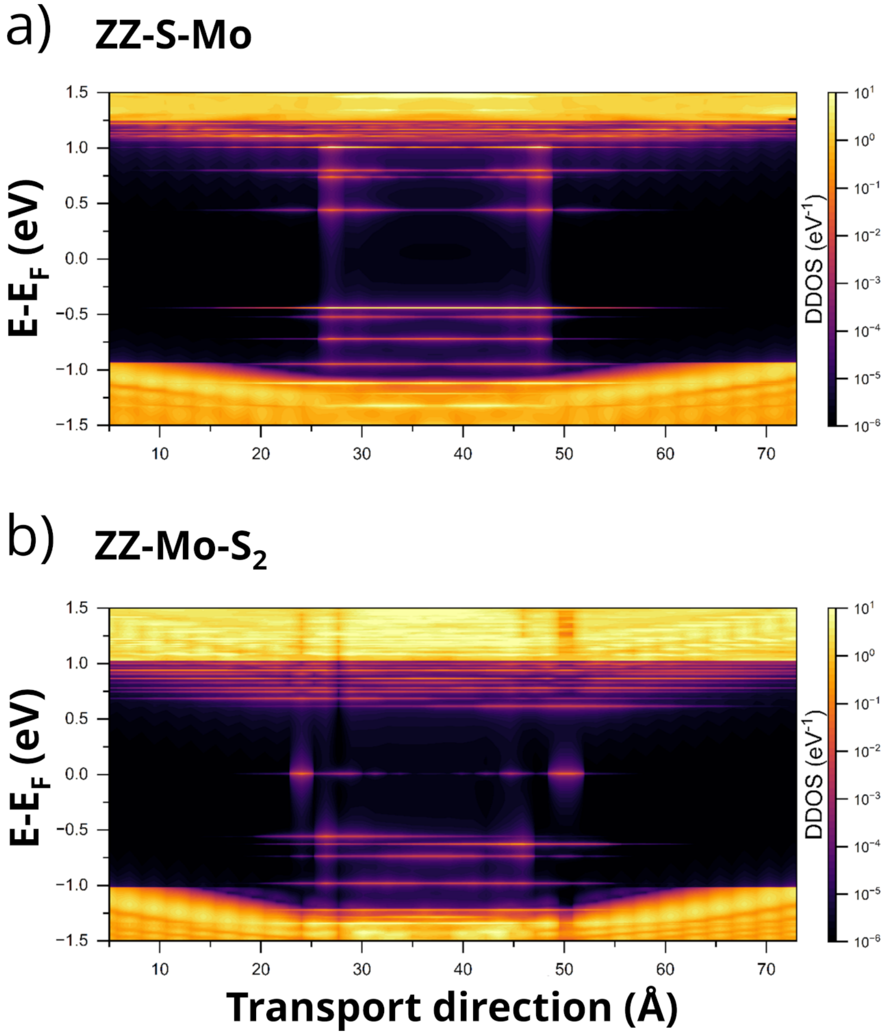

Figure. S7: Local density of states as function of device length in (a) ZZ-S-Mo, and (b) ZZ-Mo-S_2.

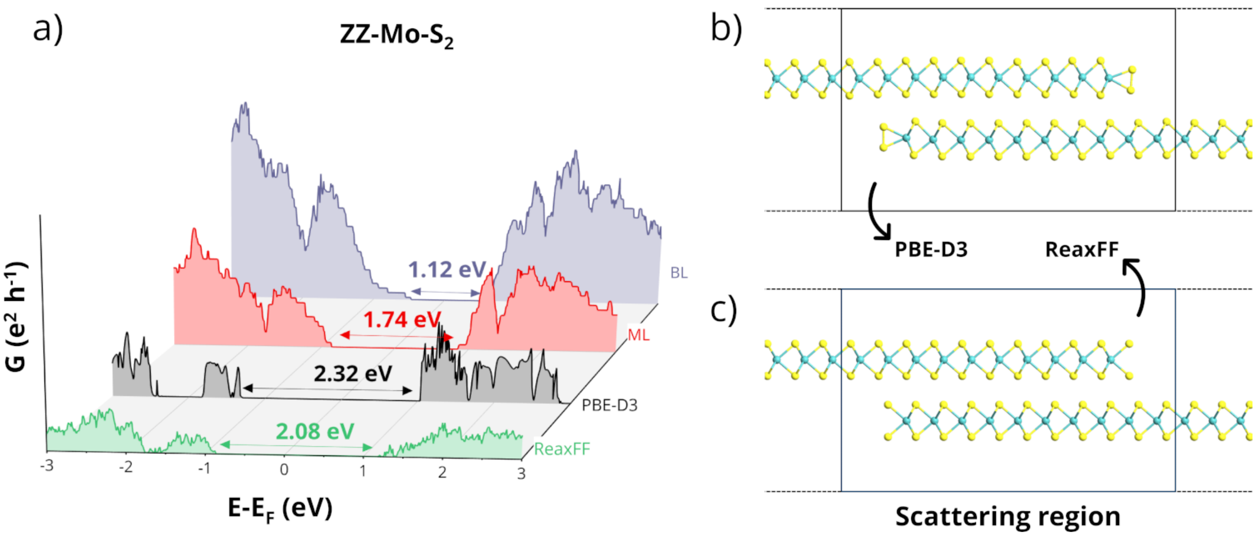

Figure. S8: (a) Conductance as function of energy for ZZ-Mo-S_2, with the scattering region fully optimized using PBE-D3 and ReaxFF methods. (b) Atomic configuration of the scattering region after optimization with PBE-D3, showing the formation of S_2 dimer at the edge. (c) Edges do not form dimer in ReaxFF optimization.
